# Synchronous Occurrence of Acute Myeloid Leukemia and Multiple Myeloma: A Case Report and Literature Review

**DOI:** 10.7759/cureus.83730

**Published:** 2025-05-08

**Authors:** Tajudeen Musbau, Bahaa Al-Bubseeree

**Affiliations:** 1 Hematology, Victoria Hospital Kirkcaldy, Kirkcaldy, GBR

**Keywords:** acute myeloid leukemia, aml, azacitidine, bone marrow, cytarabine, daunorubicin, multiple myeloma, myeloblasts, plasma cells, venetoclax

## Abstract

Multiple myeloma (MM) and acute myeloid leukemia (AML) are malignant clonal disorders with divergent lineages. It is extremely uncommon for both conditions to be diagnosed simultaneously in a patient. This case report examines the uncommon occurrence of AML alongside MM in a 60-year-old male patient. The diagnosis of AML was based on the World Health Organization benchmark, and the patient was categorized as having AML with myelodysplastic defining molecular changes. The patient completed the first cycle of induction chemotherapy with daunorubicin, cytarabine, and gemtuzumab ozogamicin but had a poor response, indicating refractory disease. A bone marrow aspiration following treatment revealed 30% blasts and 11% plasma cells. These plasma cells were CD138 positive and were not present at the time of diagnosis. Additionally, M-protein was detected in the blood. Intermediate chemotherapy with venetoclax and azacitidine was considered, as the patient was not fit for the combination of fludarabine, cytarabine, granulocyte colony-stimulating factor, and idarubicin. Furthermore, the myeloma was not treated as he was not fit for any intensive chemotherapy. The patient’s disease was refractory despite four cycles of venetoclax and azacitidine, as evident by residual blasts of 32% in the bone marrow.

## Introduction

Acute myeloid leukemia (AML) is a fast-growing myeloid malignancy marked by the clonal proliferation of immature hematopoietic stem cells, referred to as blasts, in the bone marrow. This proliferation leads to impaired erythropoiesis and megakaryopoiesis, which present quickly as bone marrow failure, in contrast to the slower progression seen in chronic leukemias [1]. AML may arise de novo, or secondary to a prior treatment, preceding hematological disorder (e.g., myelodysplastic syndrome) or germline predisposition [2]. The World Health Organization (WHO) classification classifies AML based on the presence of AML-defining genetic abnormalities, and by differentiation (i.e., for AML cases lacking defining genetic abnormalities) [2]. AML can be diagnosed and classified according to the latest WHO classification or the International Consensus Classification (ICC) [2,3]. In both classifications, bone marrow evaluation, genetic testing, and predisposing factors (e.g., prior therapy, antecedent myeloid neoplasms, and inherited genetic mutations or syndromes) are required to make a definitive AML diagnosis. However, the classifications differ in how specific subtypes of AML are categorized, and in the blast count threshold requirements for certain subtypes of AML. The 5th edition of the WHO Classification of Haematolymphoid Tumours no longer requires a blast count threshold of ≥20% for the diagnosis of AML with specific genetic abnormalities (excluding BCR:ABL1 fusion and CEBPA mutation) [2]. In the ICC, however, a blast count of ≥10% is required for diagnosing AML with specific genetic abnormalities (excluding BCR:ABL1 fusion), where a blast count of ≥20% is required [3]. Multiple myeloma (MM) is defined by the uncontrolled growth of plasma cells, leading to the production of a monoclonal antibody or immunoglobulin fragments (known as a paraprotein or M-protein) and end-organ damage. This can result in complications such as hypercalcemia, osteolytic bone disease, renal failure, anemia, and infection [4]. The International Myeloma Working Group updated criteria for the diagnosis of MM include the following: clonal plasma cells ≥10% in the bone marrow, or evidence of biopsy-confirmed bony or extramedullary plasmacytoma, and any one or additional myeloma-defining events, including signs of end-organ damage that can be linked to the underlying plasma cell proliferative disorder. These are hypercalcemia, renal insufficiency, or anemia [5,6]. AML and MM can typically occur in the same individual, but are usually observed in MM patients undergoing chemotherapeutic treatment, and AML develops during or after treatment [7]. The simultaneous presence of AML and MM in an individual is uncommon in the absence of prior chemotherapy subjection, with about 25 cases reported to date [8]. This report discusses a case in which a patient developed both AML and MM synchronously, despite having no prior exposure to chemotherapy.

## Case presentation

**Figure 1:**
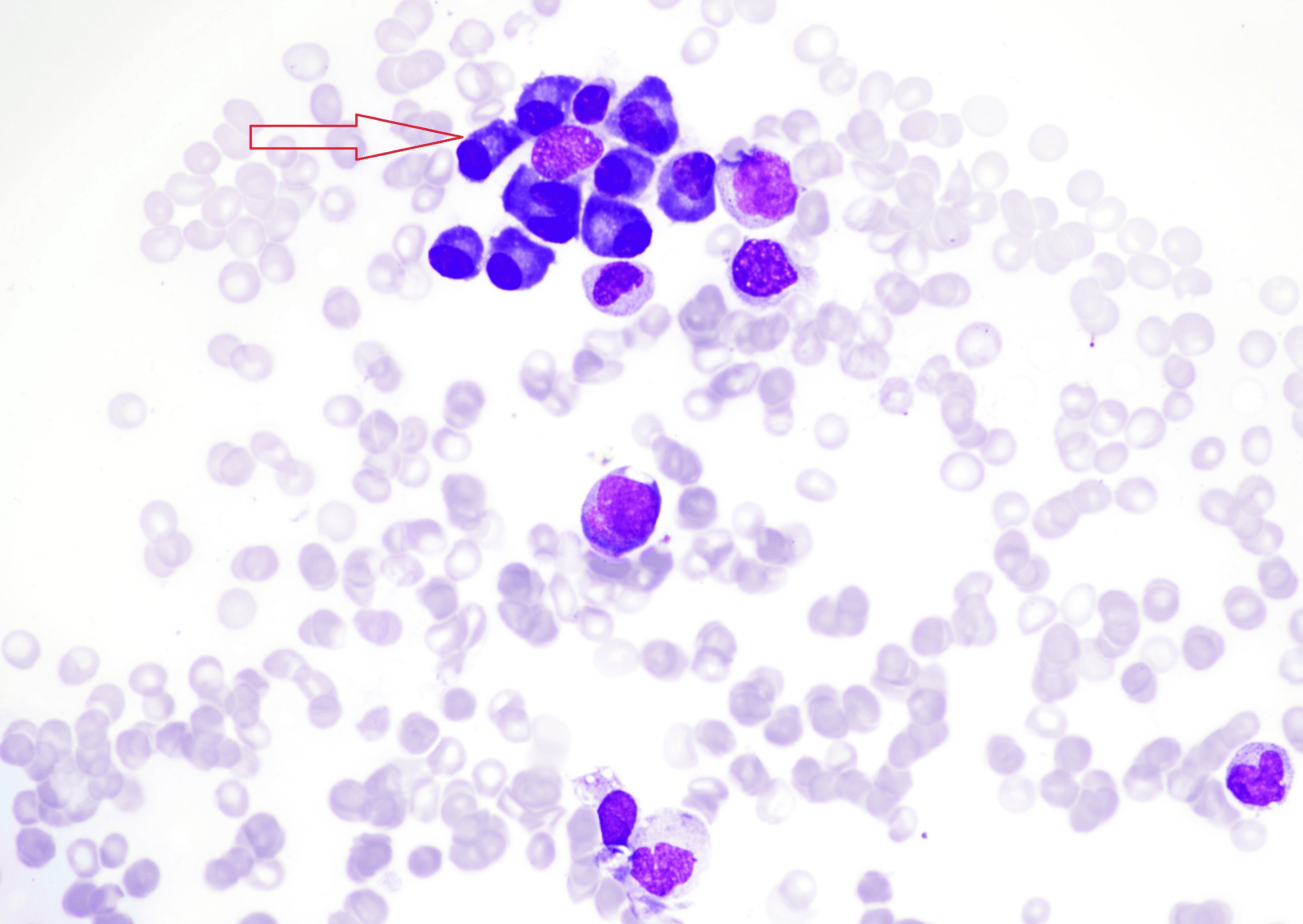
Bone marrow aspirate smear showing an increase in plasma cells with some clustering and binucleated forms in keeping with coincidental plasma cell dyscrasias.

**Figure 2:**
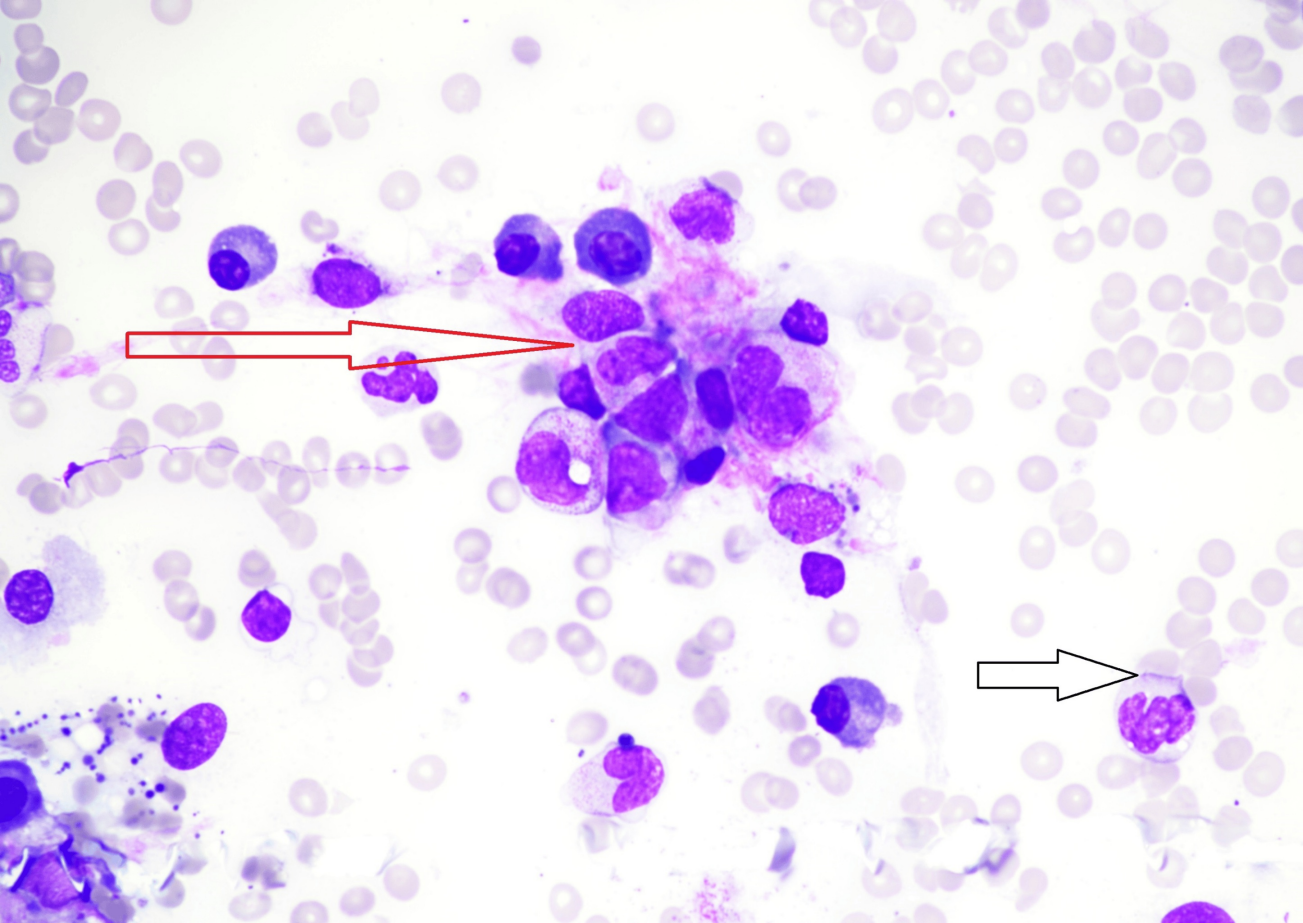
Bone marrow aspirate smear showing severely suppressed erythropoiesis with active granulopoiesis but significant dysplastic cells. Aggregates of leukemic cells (red arrow) and dysplastic neutrophil (black arrow).

**Figure 3:**
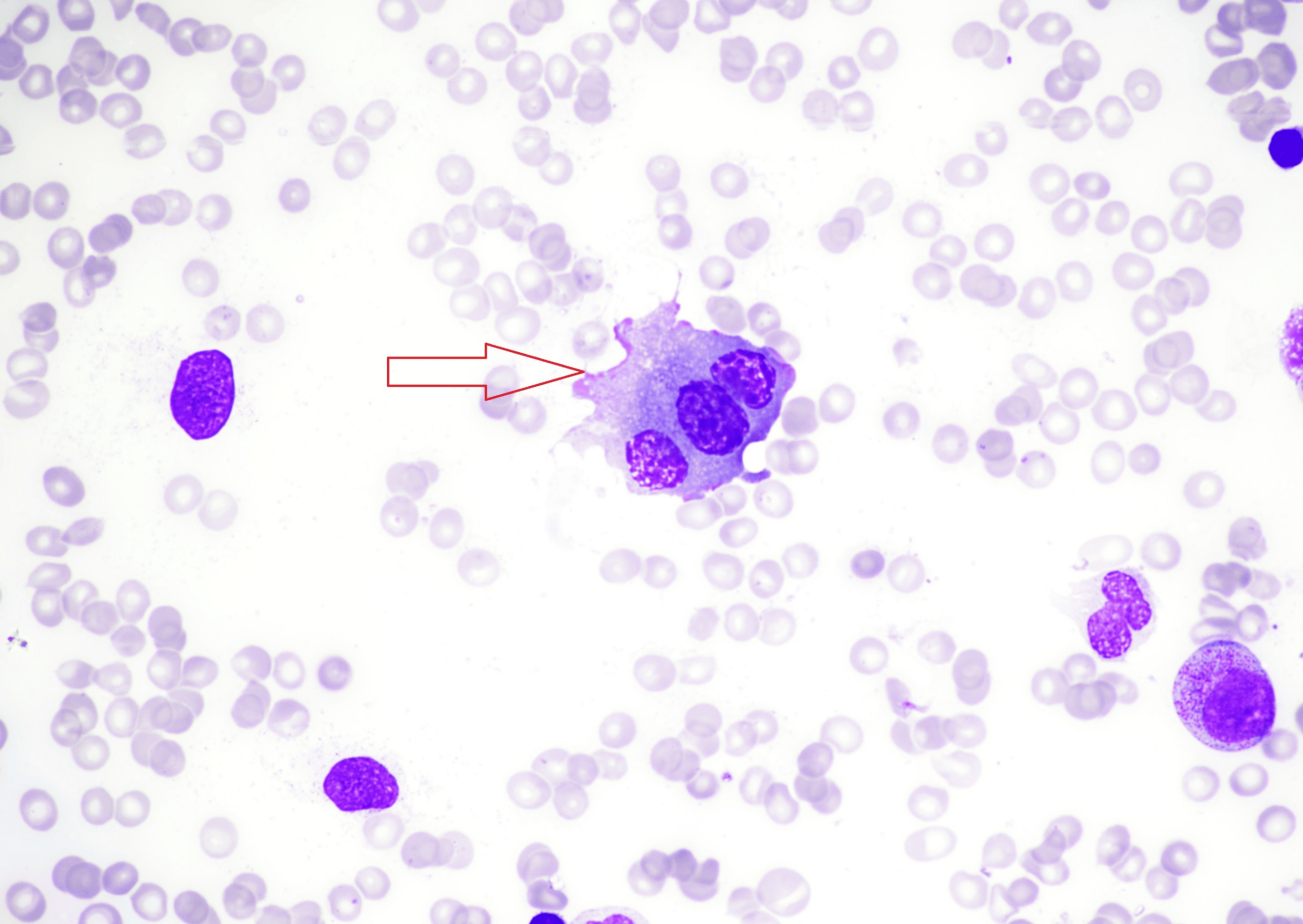
Bone marrow aspirate smear. Multinucleated plasma cell with deeply blue-staining cytoplasm and characteristic nuclear chromatin pattern.

A 60-year-old man without previous medical history initially presented to the general practitioner with tiredness and left leg pain. Blood tests (Table 1) showed leukocytosis (32,200/μL), monocytosis with an absolute monocyte count (AMC) of 6920/μL, absolute neutrophil count (ANC) of 13,840/μL, lymphocytosis of 7,560/μL, normocytic normochromic anemia (hemoglobin: 101 g/L; mean corpuscular volume: 97 FL; mean corpuscular hemoglobin: 29 pg), and thrombocytosis of 756,000/μL. Blood film showed leucoerythroblastic picture, normochromic normocytic RBC with frequent nucleated RBC dysplastic hypogranular neutrophils, monocytes, and promonocytes, myeloblasts, and thrombocytosis with frequent megakaryocytic fragments, with the blasts and blasts equivalent around 18%. Liver and kidney function were normal except for hyperkalemia of 6, and the clotting screen was also normal. He was referred to our facility immediately for additional evaluations. On arrival, there were no significant examination findings. Bone marrow aspirate showed hypercellularity with increased myeloblasts, with significant dysplastic changes, plasma cells at 1%, and myeloblasts and blast equivalent promonocytes totaling around 50-60% of marrow cells. A total of 27% of these blast cells were positive for CD34, CD117, HLADR, CD13, and CD33 (heterogeneous), and negative for CD2, CD3, CD4, CD5, CD7, CD8, CD10, CD14, CD15, CD19, CD20, CD56, and CD64. Molecular analysis showed detectable STAG2 and UTAF1 mutations; however, the karyotype was normal, and other cytogenetic abnormalities were not detected. Following diagnosis, he was started on induction chemotherapy with daunorubicin, cytarabine, and gemtuzumab ozogamicin based on the outcome of a multidisciplinary team (MDT) discussion, while awaiting the result of myeloid next-generation sequencing (NGS), which took a few weeks, and the patient was deteriorating. Regardless of treatment, the patient continued to have persistent blasts of 30% with new elevated plasma cells of 11% on bone marrow aspirate after cycle one. These plasma cells were positive for CD138 (Figures 1-3).

**Table 1:** Summary of complete blood count and serum protein electrophoresis results, along with their normal reference ranges.

Parameters	Patient	Reference
Hemoglobin	101	130-170 g/L
White cell count	32,200	4000-10,000/μL
Platelet count	756,000	150,000-410,000/μL
Neutrophil count	13,840	2000-7000/μL
Monocyte count	6,920	200-1000/μL
Lymphocyte count	7,560	1000-3000/μL
Mean corpuscular volume	97	83-101 FL
Mean corpuscular hemoglobin	29	27-32 pg
Kappa light chain	97.3	3.3-19.4 mg/L
Lambda light chain	43.3	5.7-6.3 mg/L
Kappa/lambda ratio	2.25	0.26-1.65

Further investigations revealed IgG paraproteinemia, kappa light chains of 97.3 mg/L, lambda light chains of 43.3 mg/L, and a kappa/lambda ratio of 2.25. The paraproteins were not checked at admission, making it difficult to compare with the baseline and determine if they had decreased due to chemotherapy. Due to refractoriness of his AML, new diagnosis of MM, and his fitness for intensive chemotherapy, intermediate chemotherapy with venetoclax and azacitidine (Ven/Aza) was considered. Subsequently, he received four cycles of venetoclax and azacitidine with ongoing refractory AML (persistent blasts count of 32%). Due to limited therapeutic options and prolonged cytopenia, he was counseled for best supportive care and symptom control with regular pancytopenia management.

## Discussion

The relationship between AML and MM is generally regarded as a complication resulting from chemotherapy. However, it can also occur without prior chemotherapy treatment [9]. Several potential etiopathogeneses are suggested to describe the concurrent development of the dual cancers. These encompass a dysfunction of multipotent stem cells, submission to shared surrounding risk factors, and frequent infections in MM patients due to hypogammaglobulinemia, which could lead to the formation of clones of leukemic cells [10]. Another possibility is the gradual progression of MM that results in diminished immune surveillance, allowing emerging leukemic clones to evade detection by the immune system [11,12]. In the current case, the initial percentage of plasma cells at diagnosis was 1%, which could have been obscured by the presence of circulating blasts in the bone marrow or the early stages of evolving myeloma. An increase in the plasma cell population became more noticeable as the number of blast cells decreased after chemotherapy. The concomitant occurrence of these two malignancies in a patient without prior chemotherapy was reported by Kim et al. [13]. This shows the emerging occurrence of both diseases without significant exposure to chemotherapeutic agents [13].

The survival time from diagnosis has been estimated to be from a few weeks to 15 months, with IgG kappa being the most frequent paraprotein identified in patients with concurrent MM and AML [10]. These were consistent with the findings in our patient, who is still alive nine months after starting treatment. In 2003, Luca and Almanaseer conducted flow cytometry and exhibited the presence of blasts, which were positive for CD14, CD33, and human leukocyte antigen (HLA)-DR, with negative CD45 for plasma cells [14]. A case of concurrent AML and MM kappa-type paraprotein was reported by Kim et al. [13]. Bone marrow cells analyzed through immunohistochemistry revealed the presence of plasma cells positive for CD138 with kappa light chain restriction, while myeloblasts were positive for CD34 and CD117. In addition, the existence of two well-defined malignant plasma cells and myeloblasts was confirmed on flow cytometry [13].

The prognosis is still very poor, as there is no established standard treatment due to the rarity of the condition. Patients are commonly treated with AML therapies, given the more aggressive nature of AML, and the anthracycline drug class appears to be successful for treating MM [10]. Raz and Polliack reported a case of a 68-year-old man who developed both AML and MM simultaneously. He was successfully treated with a combination of vincristine, cyclophosphamide, melphalan, and prednisolone, which led to the disappearance of monoblasts and a reduction in serum paraprotein levels [15]. Kim et al. also reported a case of a 51-year-old man, previously healthy and in good condition, who was simultaneously diagnosed with myeloma and AML. The treatment of both malignancies was successful through allogeneic stem cell transplantation after induction with cytarabine and idarubicin alongside bortezomib [13]. The combination of bortezomib, cytarabine, aclarubicin, and granulocyte colony-stimulating factor (CAG) has shown significant curative effects in elderly patients who are not candidates for allogeneic stem cell transplantation. In this case, the patient achieved and sustained remission for over six months [9]. In the present case, the AML was refractory to treatment, and myeloma was left untreated as the patient was not fit for any intensive chemotherapy. Furthermore, due to limited therapeutic options and prolonged cytopenia, he was counseled for best supportive care and symptom control with regular pancytopenia management.

## Conclusions

The concomitant presence of AML and MM in an individual with no prior cytotoxic exposure or treatment is exceptionally uncommon. Patients diagnosed with both hematological malignancies often have a poor prognosis, and an appropriate intervention is yet to be established.
